# Decision tree-based approach to robust Parkinson's disease subtyping using clinical data of the Michael J. Fox Foundation LRRK2 cross-sectional study

**DOI:** 10.3389/frai.2025.1668206

**Published:** 2025-11-21

**Authors:** Soumyadeep Roy, Stefanie Krähe, Michael Marschollek, Helge Frieling, Niloy Ganguly, Dominik Wolff

**Affiliations:** 1 Department of Computer Science and Engineering, Indian Institute of Technology Kharagpur, Kharagpur, India; 2 L3S Research Center, Leibniz University Hannover, Hannover, Germany; 3 Hannover Medical School, Hannover Unified Biobank (HUB), Hannover, Germany; 4 Peter L. Reichertz Institute for Medical Informatics of TU Braunschweig and Hannover Medical School, Hannover Medical School, Hannover, Germany; 5 Department of Psychiatry and Psychotherapy, Hannover Medical School, Hannover, Germany

**Keywords:** Parkinson's disease, data-driven subtyping, artificial intelligence, age of onset, decision tree classifier, clinical characterization, diagnostic markers

## Abstract

Parkinson's Disease (PD) is a neurodegenerative disorder with high heterogeneity in clinical symptoms, progression course, treatment response, and genetic factors. Thus, PD subtyping aims to enhance understanding of disease mechanisms and helps to facilitate targeted interventions or treatment regimens. Data-driven PD subtyping is typically done using cluster analysis. Still, such studies face difficulty from widespread adoption in clinical practice due to the following issues: (i) results are quite sensitive to study design, and actual subtype rules are not reasonably interpretable; (ii) results are not robustly replicable across multiple datasets, and most studies focus on a single dataset. This paper aims to identify novel PD subtypes using an interpretable decision-tree-based method that is robustly reproducible in an independent PD cohort. We first train a decision tree classifier on an LRRK2 dataset to determine whether a patient has early onset or late onset PD. By tracing back from the leaves of the learned decision tree subtyping rules are established. The independent MDS dataset is used for external validation, after mapping features between the two datasets. We finally obtained six novel subtypes that are clinically consistent and sufficiently large across both training and external validation datasets. Finally, a clinical characterization study showed that the following clinical features may be the most important diagnostic markers for our six detected subtypes: (i) persistent asymmetry affecting the side of onset most, (ii) clinical course of 10 years or more, and (iii) postural instability not caused by other dysfunction. The subtypes identified in our study may provide relevant guidance for prognosis and therapeutic strategies. An early onset subtype (E4) can be linked to a comparatively favorable prognosis. In contrast, the mixed onset subtypes (M3 and M7) may predict faster functional decline, suggesting that patients in these groups could benefit from intensified supportive measures. One late onset subtype (L1) seems to have a more benign course, while the other two (L2 and L4) are connected with predictors of reduced quality of life and increased care dependency.

## 1 Introduction

Parkinson's disease (PD) is a progressive neurodegenerative disease clinically characterized by a broad spectrum of motor and non-motor manifestations (Armstrong and Okun, 2020). In addition, PD is associated with wide variability in clinical manifestations, progression course, treatment response, underlying genetic mechanisms, biomarker readouts, and pathology (Brendel et al., 2021). Personalized treatment in PD with a focus on the individual is also being explored (Titova and Chaudhuri, 2017). Thus, researchers focus on identifying symptom-based (both motor and non-motor) PD subtypes and genetic subtypes, such as those associated with mutations in glucocerebrosidase (GBA1) or leucine-rich repeat kinase 2 (LRRK2) (Sardi et al., 2018). Patients with mutations in the LRRK2 gene are more likely to experience tremors, respond to levodopa, and are less likely to develop cognitive impairment and hyposmia (Huang et al., 2007; Kestenbaum and Alcalay, 2017).

Using the *age of onset* for categorizing PD patients is a well-accepted subtyping solution due to its simplicity and clinical applicability (Qian and Huang, 2019). The *age of onset* (AO) is defined as the age at which the onset of motor symptoms starts (Mehanna et al., 2022). Despite established differences between juvenile Parkinsonism, early onset PD (EOPD), and late onset PD, patients with an age of onset of 50 years or less represent only 5%–10% of the PD population (Bertucci Filho et al., 2007), while late onset PD remains highly heterogeneous in terms of symptoms and treatment outcomes (Qian and Huang, 2019). Furthermore, there is no consensus on the age limit to distinguish between EOPD and late onset PD. In literature, various cut-offs have been used, such as 50, 55, and 60 years of age, highlighting the arbitrary nature of this subtyping method (Wickremaratchi et al., 2011). Mehanna et al. (2022) investigated this exact issue and finally defined EOPD as AO greater than 21 years and less than 50 years. We utilize this formulation and define late onset PD (LOPD) as AO greater than 50 years. Early onset PD patients (EOPD) usually have a better prognosis, with slower motor and cognitive progression (Schrag and Schott, 2006) compared to late onset PD. A study found that the advanced stages of PD may be similar for both subtypes and are characterized by an increasingly rapid decline in motor and cognitive domains. It concluded that the age of onset only primarily influenced the progression rate in the early stages of the disease (Kempster et al., 2010). Despite this, EOPD patients are likely to experience early motor difficulties, such as disabling dyskinesias, often painful dystonia, and possibly severe motor fluctuations (Salawu, 2012; Werner et al., 2010). Most empirical PD subtyping studies solely focus on motor symptoms. Nevertheless, non-motor symptoms (e.g., cognitive impairment, autonomic dysfunction, mood disorders) are increasingly recognized as distinguishing factors for early and late onset PD as well as subtyping (Ramirez-Zamora, 2022).

However, traditional PD subtyping studies are based on empirical analysis of (often dichotomous) cohort studies and thus focus only on a small aspect of PD at a time (Brendel et al., 2021). Dadu et al. (2022) state that intuitive dichotomous separations, such as *early onset vs. late onset* disease, *slowly-progressing “benign” vs. fast-progressing “malignant” subtypes, PD with or without dementia*, or based on the most prominent clinical signs into a *tremor-dominant vs. a postural instability with gait disorder* subtype, do not truthfully represent the quantitative, complex, and inter-related nature of the disease's clinical features. To overcome such problems, Ramirez-Zamora (2022) suggested transitioning to multi-dimensional data-driven approaches, which enable a more holistic view of the disease as different features, such as motor and non-motor symptoms, are analyzed in combination.

In recent studies, data-driven patient subtyping is formulated as a clustering problem (Zhang et al., 2019; Krishnagopal et al., 2020; Brendel et al., 2021; Rodriguez-Sanchez et al., 2021; Markello et al., 2021). However, in the context of PD subtyping, cluster analysis-based studies have various limitations: (i) Cluster analysis is a highly sensitive statistical method. Here, additional features exceeding the essential properties result in models with low reproducibility and limited statistical validity (Qian and Huang, 2019). Qian and Huang (2019) thus recommended that subtyping should be based on the overall features of PD, for example, by relying on MDS-UPDRS-I, II, III at a drug-naive state. (ii) Different clustering studies carried out on the same dataset do not necessarily produce subtypes that overlap to a large extent. For the Parkinson's Progression Markers Initiative (PPMI) cohort, the overlap was only 56%. This highlights that clustering approaches are highly sensitive to the study design (Fereshtehnejad and Postuma, 2017), like cohort composition and feature representation (Qian and Huang, 2019). Another major issue with recent data-driven PD subtyping studies is a lack of external validity, i.e. evaluating the found subtypes in an independent cohort as well as transferring results to clinical practice (Qian and Huang, 2019; Mestre et al., 2021).

This paper aims to identify novel and meaningful PD subtypes in an interpretable manner, as well as to validate these subtypes in an independent PD cohort. We first develop an interpretable data-driven decision tree-based PD subtyping method. We train a decision tree model on the LRRK2 dataset (Michael J. Fox foundation for Parkinson's Research, 2023) for the binary classification task of determining whether a PD patient has early or late onset PD. The leaves of the learned decision tree are considered candidate subtypes, which are then filtered in multiple stages to ensure their validity. After filtering, the remaining subtypes are validated in an independent cohort [Movement Disorder Society (MDS) dataset (Chaudhuri et al., 2020)] to determine whether the clinical characteristics of a given PD subtype are consistent across different PD cohorts. We further perform an in-depth characterization study of these automatically learned subtypes to identify potential markers for PD and their clinical significance. Identification of these predictive markers paves the way toward early therapeutic intervention in neurodegenerative diseases (Mestre et al., 2021). Our decision tree-based approach addresses most of the issues of clustering-based subtyping. First, subtyping rules derived from a decision tree directly reflect the features and their cut-off values that were responsible for the classification, making them more human-interpretable compared to clustering results. The learned rules can be reviewed by a medical expert at any stage of the training process, allowing for early feedback and adjustments as needed. Second, we performed 10-fold cross-validation, ensuring a high intra-dataset validity of the found subtypes as well as external validation in an independent cohort for inter-dataset validity. Third, use the age of onset as a guiding feature. It is known to influence disease manifestation (Qian and Huang, 2019) in various ways, such as progression speed (Alves et al., 2005; Jellinger, 2018). By targeting it as a weak classification goal while allowing heterogeneous subtypes, this approach integrates this knowledge without directly using it as a feature. Fourth, we enforce the decision tree to focus on the most distinguishing features of potential subtypes by limiting its depth.

We develop a decision tree-based binary classification Machine Learning (ML) model to predict whether a person has early onset or late onset PD based on clinical data. Here, we address the known issues of classical clustering-based subtyping approaches in the following manner: (i) The clinical features we use are primarily from the MDS-Unified Parkinson's Disease Rating Scale (MDS-UPDRS), which ensures that our proposed subtypes are based on a holistic range of features. (ii) Instead of a fully unsupervised approach, as done in the case of clustering, we use the age of onset as the target label for our decision tree-based approach. It may be considered equivalent to a form of guided clustering. We transform the learned decision tree rules into granular patient subtype conditions. These form the candidate novel subtypes that are then subjected to a strict filtering step to form the proposed novel subtypes.

Our aim is not just identifying novel PD subtypes, but they should also be robust, i.e., they are valid in a second cohort. Therefore, the strict requirement of an external validation dataset guided our patient subset selection process from the LRRK2 dataset. After performing a mapping of features, we validate these derived subtypes by using the equivalent features between LRRK2 and MDS datasets, primarily from the MDS-UPDRS rating scale. Unfortunately, the LRRK2 data were primarily derived from the Tunisian population due to the requirement of having overlapping clinical features, specifically MDS-UPDRS scores, between the primary dataset (MJF LRRK2 Cross-sectional Study) and the external validation dataset (MDS study). While this approach led to a narrow geographic representation in the LRRK2 dataset, it ensured that the identified subtypes were not specific to a single population and could be validated in an independent general PD cohort. The limitations of this patient selection process are acknowledged and discussed in more detail in the “Section 3.” Future work will aim to expand the analysis to more diverse PD populations, such as the PPMI dataset of the Michael J. Fox Foundation.

## 2 Results

We first describe the predictive performance of the decision tree-based PD classification model. Next, we describe the six novel subtypes that we identify.

### 2.1 Decision tree-based PD classification model

We develop a decision tree model for the binary classification task of predicting patients as early or late onset PD. We use the leaves of the learned decision tree as candidate PD subtypes for further investigation. Based on 10-fold cross-validation, we obtain the best parameter value of *max depth* as 8 and *minimum samples per leaf* as 12. We achieve a validation accuracy of 0.68, a test accuracy of 0.662, and a test AUROC score of 0.691. Two issues arising due to problem setting negatively impact the model performance: (i) high-class imbalance in training data where early onset PD patients are in the minority, (ii) restriction on the *minimum sample per leaf* parameter (at least 10) to obtain sufficiently large subtypes (i.e., subtypes must have at least 10 unique PD patients). This allows us to analyze the biological characteristics of each subtype with reasonable generalizability. Smaller subtypes would also result in insufficient positive matches with the external validation dataset, which will be discussed in the next subsection. We observe that the model's test accuracy increases significantly to 0.74 when the minimum sample per leaf is lowered to 4. However, such a small value for *minimum sample per leaf* will lead to candidate subtypes that will be smaller, indicating that the currently proposed subtypes may theoretically be further subdivided.

**Figure 1:**
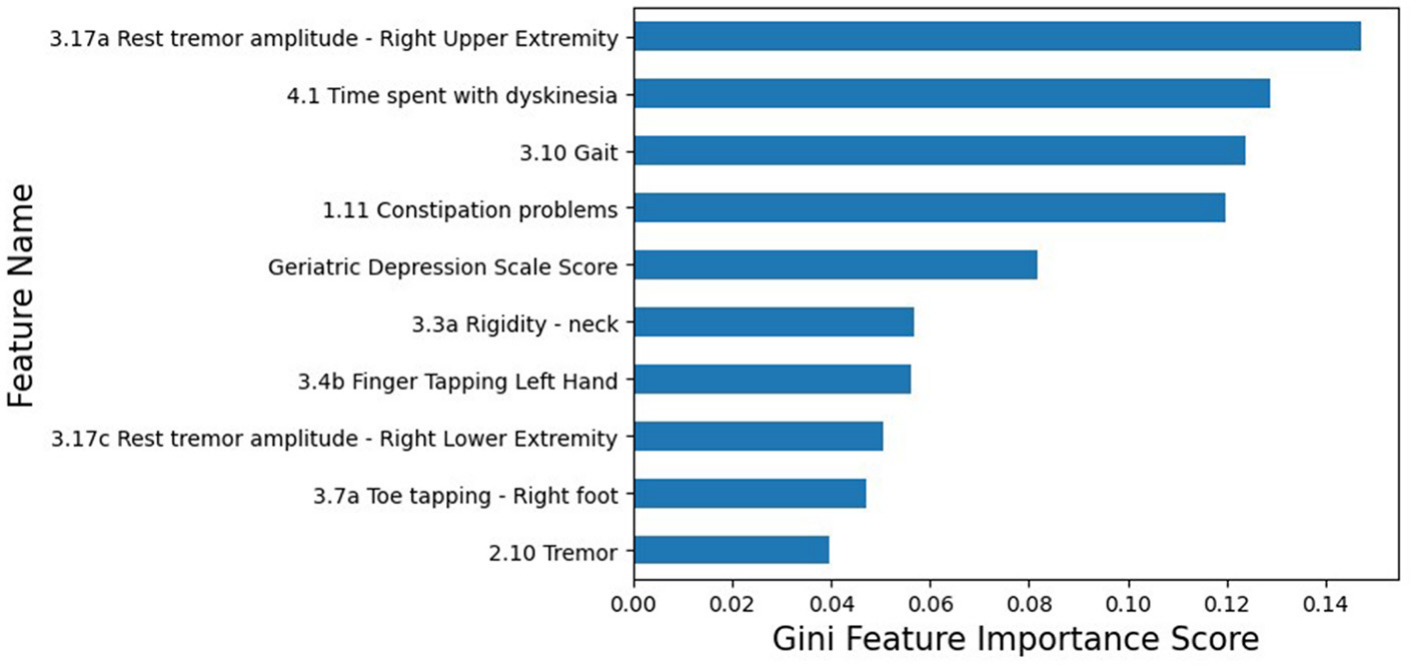
Gini feature importance scores in descending order for our learned decision tree-based PD binary classification model. The Gini importance score of a feature is computed as the (normalized) total reduction of the criterion brought by that feature. Except for the Geriatric Depression Scale Score, all the remaining features belong to the MDS-UPDRS Rating Scale; we also report the question ID to easily identify the feature characteristics from the MDS-UPDRS Rating Scale.

We assign a late onset PD patient a label of 1 and the early onset PD patient a label of 0 for the purpose of binary classification. The precision is 0.878, and the recall is 0.632. We observe that our decision tree-based PD classification model achieves high precision for late onset PD patients and low precision for early onset PD patients. Figure 1 shows the feature importance scores in decreasing order of the decision tree model. We observe that eight of the top 10 most important features are motor symptoms, while the non-motor symptoms (both within the top five ranks) are constipation problems and Geriatric Depression Scale Score.

### 2.2 Filtering of candidate subtypes to form final subtypes

We consider each leaf of the learned decision tree as a candidate subtype. We perform two stages of filtering based on sufficient representation of the subtypes in training (LRRK2 dataset) and external validation (MDS) dataset, as well as precision of subtypes between these two datasets (the filtering steps are further explained in the Section 4); we will next explain the filtering step-related results. We adopt the age of PD onset (AO)-based subtyping criteria proposed by Mehanna et al. (2022) for categorizing each PD patient as EOPD or LOPD, where it is deduced that EOPD patients have an AO greater than 21 years and less than 50 years, while LOPD patients have an AO greater than 50 years. We use the same criteria for identifying EOPD and LOPD patients in both LRRK2 and MDS data. We categorize a candidate subtype as *primarily early onset* when most of them are EOPD patients (sufficiently skewed, with Gini impurity ≤ 0.4), *primarily late onset* when most of them are LOPD patients, and *mixed onset* when there is no clear majority between EOPD and LOPD patients (Gini impurity >0.4).

We perform an in-depth feature and subtype mapping with the MDS dataset (an independent PD cohort of 402 PD patients), which is used to check the external validity of the candidate subtypes to validate the novel PD subtypes identified in our work. Table 1 shows the list of candidate subtypes obtained from the learned decision tree that have at least one positive match with the MDS dataset. We impose the criteria that a well-validated subtype should have at least 5% positive matches with the same age-onset-based subtype in the MDS data (used for external validity). For example, *subtype E4*, a primarily *early onset* subtype, should match with at least 5% of EOPD patients of MDS data for successful external validation. Therefore, the minimum number of patients in the *primarily early onset, primarily late onset*, and *mixed onset* candidate subtypes need to be 4, 17, and 21, respectively. The six candidate subtypes that satisfy the criteria are *subtypes E4, L1, L2, L4, M3*, and *M7*. The precision-based filtering is then applied to only the *early onset* and *late onset* subtypes among these six candidate subtypes, and we conclude from Table 2 that all of them satisfy the criteria, which further strengthens the reliability of our determined final subtype class (*early onset* or *late onset*). Thus, these six subtypes (*E4, L1, L2, L4, M3*, and *M7*) are considered our (proposed) final subtypes.

**Table 1:** Subtype selection from the candidate subtypes based on external validation data of MDS.

**Subtype Id**	**Tree depth**	**True positive**	**Overall positives**	**True positive (in %)**	**False positive**	**Overall negatives**	**False positive (in %)**
E1	3	2	68	2.9	9	321	2.8
E2	4	3	68	4.4	23	321	**7.2**
E3	3	3	68	4.4	5	321	1.6
**E4**	4	20	68	**29.4**	50	321	**15.6**
**L1**	6	39	321	**12.2**	6	68	**8.8**
**L2**	5	29	321	**9.0**	1	68	1.5
L3	3	7	321	2.2	0	68	0.0
**L4**	5	25	321	**7.8**	4	68	**5.9**
L5	5	7	321	2.2	0	68	0.0
L6	7	12	321	3.7	2	68	2.9
M1	5	8	402	2.0	–	–	–
M2	3	2	402	0.5	–	–	–
**M3**	5	47	402	**11.7**	–	–	–
M4	6	2	402	0.5	–	–	–
M5	8	5	402	1.2	–	–	–
M6	4	16	402	4.0	–	–	–
**M7**	3	38	402	**9.5**	–	–	–

Subtype IDs starting with the characters “E,” “M,” and “L” refer to primarily early onset, mixed onset, and primarily late onset PD subtypes, respectively. “Overall positives” is the total number of patients for the relevant age-of-onset, i.e. 68, 321, and 402 for EOPD, LOPD, and all patients in the MDS dataset, respectively. The candidate subtypes that pass the 5% positive match with MDS data criteria are bolded. Since a mixed onset candidate subtype has no clear majority between EOPD and LOPD patients, we conclude that such subtypes may be independent of age of onset (i.e., it cannot be defined in terms of age of PD onset). Therefore, we compute the positive match with the entire MDS dataset and keep its threshold as 21 (5% of 402 = 20.1).

**Table 2:** Precision-based filtering criteria for *early onset* and *late onset* candidate subtypes.

**Subtype Id**	**EOPD matches**	**LOPD matches**	**Precision**	**Precision threshold**
E4	20	50	0.286	0.175
L1	6	39	0.867	0.825
L2	1	29	0.967	0.825
L4	4	25	0.862	0.825

The precision thresholds are 0.825 and 0.175 for primarily late onset PD (LOPD) and primarily early onset PD (EOPD), respectively.

### 2.3 Characterization study of novel PD subtypes

**Figure 2:**
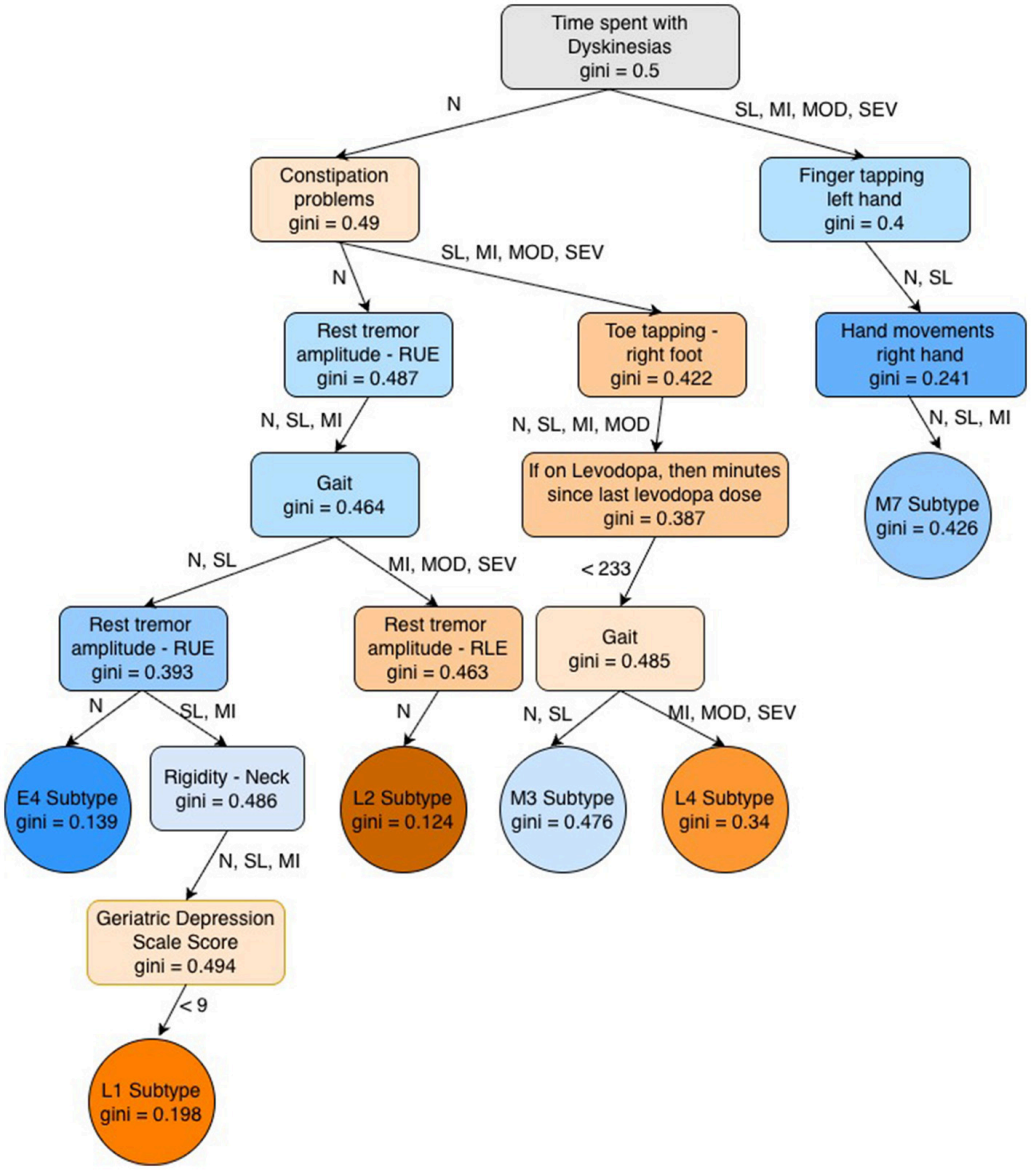
Feature conditions used to define final subtypes. Subtype IDs starting with the characters “E,” “M,” and “L” refer to primarily early onset, mixed onset, and primarily late onset PD subtypes, respectively. Primarily early and late-onset subtypes are highlighted by blue and orange color respectively. Color darkness is proportional to the degree of skewness; a lower gini score represents higher skewness and therefore has a darker color. Most of the features belong to the MDS-UPDRS Rating Scale and contain ordinal values in the order of *Normal (N), Slight (SL), Mild (MI), Moderate (MOD)*, and *Severe (SEV)*. *RUE* stands for *Right Upper Extremity* and *RLE* for *Right Lower Extremity*.

**Figure 3:**
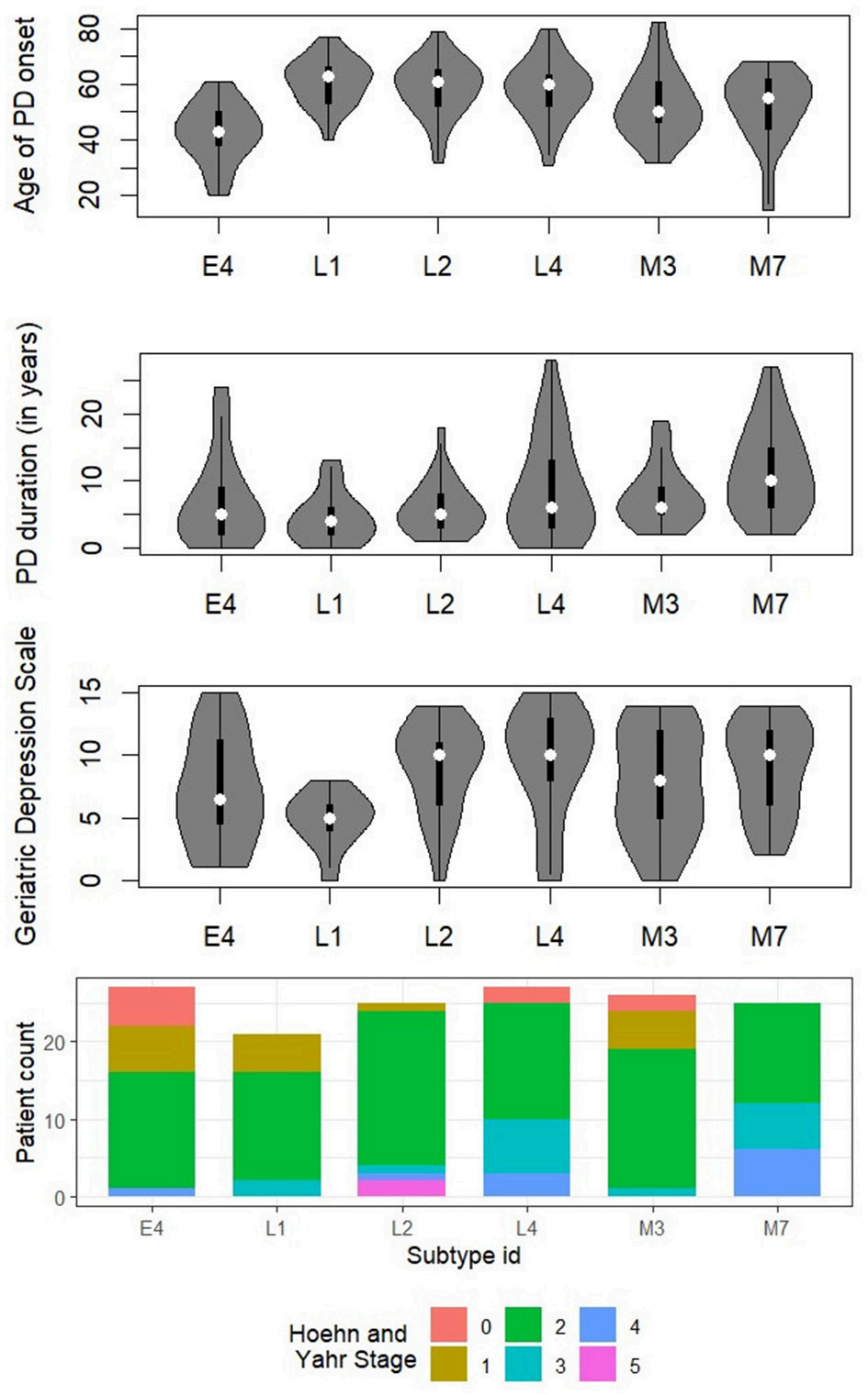
Comparison among the identified novel subtypes in terms of age of PD onset, PD duration, Geriatric Depression Scale (GDS15SCORE), and Hoehn and Yahr Stage. Subtype IDs starting with the characters “E,” “M,” and “L” refer to primarily early onset, mixed onset, and primarily late onset PD subtypes, respectively. In a violin plot, the median is shown as a white dot, and the interquartile range (IQR) is shown as a black bar in the center of the violin plot, where the lower end and upper end represent the first and third quartiles, respectively. The lower and upper adjacent values are shown as the bottom and top end of the black line stretched from the black bar, respectively. PD duration is computed as the difference in years between a patient's present age and age of PD onset (in the case of both LRRK2 and MDS data). For clinical purposes, a GDS15SCORE of greater than five is suggestive of depression and should warrant a follow-up interview, whereas a GDS15SCORE of >10 is almost always depression.

The final subtypes (obtained after multiple filtering stages) are described in terms of feature conditions in Figure 2, and demographic details for each subtype are provided in Table 3. Figure 3 compares the distribution among the final subtypes in terms of age of PD onset, PD duration, and the Hoehn and Yahr Stage (a commonly used scale to measure the nature of the progression of the symptoms of Parkinson's disease). We observe that the majority of patients across the final subtypes have *Bilateral involvement without impairment of balance*, which corresponds to Hoehn and Yahr Stage 2. Subtypes L4 and M7 comprise a large portion of patients with a more severe form of PD, as concluded based on the majority of patients with Hoehn and Yahr Stages 3 and 4 (3: mild to moderate involvement, 4: severe disability, still able to walk or stand unassisted). Subtypes L2, L4, and M7 subtypes can also be associated with almost always having depression since their median values in the Geriatric Depression Scale Score are greater than 10 (see Figure 3).

**Table 3:** Comparison of clinical characteristics of detected subtypes based on Diagnostic Check Sheet features on LRRK2 data.

**Features**	**Description**	**E4**	**M3**	**M7**	**L1**	**L2**	**L4**	**Overall**
Patient count		27	26	25	21	25	27	397
Assessment age (present age)	Mean ± s.d.	49.44 ± 10.46	60.5 ± 10.38	63.04 ± 10.74	65.29 ± 9.37	64.4 ± 9.98	66.78 ± 9.8	62.53 ± 12.06
Age of PD onset	Mean ± s.d.	42.63 ± 10.48	52.77 ± 12.17	51.44 ± 13.29	60.86 ± 8.58	58.6 ± 10.28	57.89 ± 10.94	54.50 ± 12.97
Gender	Male–female	13 − 14	17 − 9	18 − 7	13 − 8	11 − 14	15 − 12	214 − 183
PD duration	Mean assessment age minus mean age of PD onset (in years)	6.81	7.73	11.6	4.43	5.8	8.89	8.03
lrrk2sub	Does subject carry LRRK2 mutation	0.333	0.462	0.24	0.619	0.36	0.481	0.403
Diagnostic check sheet features								
DCBRADY	Bradykinesia	1.0	1.0	1.0	1.0	1.0	1.0	0.997
DCRIGID	Rigidity	0.852	0.769	**0.96**	0.905	0.84	0.926	0.856
DCRTREM	Resting tremor	**0.556**	0.846	0.88	0.857	0.76	0.852	0.826
MPTPEXP	MPTP exposure	0.0	0.0	0.0	0.0	0.0	0.037	0.003
BABINSKI	Babinski sign	0.0	0.0	0.0	0.0	0.0	0.037	0.005
NELEONSX	Neuroleptic treatment at onset of symptoms	0.0	0.0	0.0	0.0	0.0	0.074	0.01
AUNOERLY	If experienced severe autonomic involvement was it early in disease	0.0	0.077	0.0	0.0	0.0	0.074	0.018
RTRMHZ	If resting tremor is it 4–6 Hx	**0.704**	1.0	1.0	1.0	0.88	1.0	0.937
DCPRGDIS	Progressive Disorder	0.889	1.0	1.0	0.952	1.0	1.0	0.972
DCASYMM	Persistent asymmetry affecting the side of onset most	**0.556**	**0.808**	**0.88**	**0.524**	**0.56**	0.704	0.698
DCLDOPCH	Severe levodopa-induced chorea	0.0	0.038	0.12	0.0	0.0	0.037	0.045
DCPD10Y	Hx of clinical course of 10 years or more	0.185	0.269	**0.48**	**0.095**	**0.12**	0.296	0.239
HXENCEPH	Hx of definite encephalitis	0.0	0.0	0.0	0.0	0.0	0.037	0.003
DCPOSINS	Hx or present at visit with postural instability not caused by other dysfunction	0.148	**0.115**	**0.44**	**0.048**	0.16	0.148	0.232
STRKSTPD	Hx repeated strokes with stepwise progression of Parkinsonian features	0.0	0.0	0.0	0.0	0.0	0.037	0.005
SUSTRMS	Sustained remission	0.0	0.0	0.0	0.0	0.0	0.074	0.005
OGC	Oculogyric crisis	0.0	0.0	0.0	0.0	0.0	0.037	0.003
SGPSVSAC	Supranuclear gaze palsy or slowing of vertical saccades	0.0	0.0	0.0	0.0	0.0	0.074	0.008
CERESIGN	Cerebellar signs	0.0	0.0	0.0	0.0	0.0	0.037	0.003
HXUNIL3Y	Hx of strictly unilateral features after 3 years	0.0	0.077	0.0	0.0	0.0	0.037	0.018

The values under *Diagnostic Check Sheet* are computed as the ratio of the number of patients with a positive score for the given feature and the total patient count (first row) of the given subtype. For a given feature of the *Diagnostic Check Sheet* (one row), we highlight the value of a subtype if the difference with the corresponding “Overall” column value is greater than 0.1. We highlight a feature as green if it globally differentiates 50% or more of our final subtypes (i.e., three or more in our case), whereas if a feature distinguishes less than three of our final subtypes, the corresponding feature row is highlighted by yellow. Subtype IDs starting with the characters “E,” “M,” and “L” refer to primarily early onset, mixed onset, and primarily late onset PD subtypes, respectively.

We discovered one primarily early onset PD subtype (E4), two mixed onset PD subtypes (M3 and M7), and three primarily late onset PD subtypes (L1, L2, and L4). We observe that the M3 subtype is skewed toward late onset PD patients in the external validation dataset (MDS), while subtypes *E4* (precision = 0.286) and *M7* (precision = 0.353) subtypes are skewed toward patients with early onset PD in the MDS dataset. All the subtypes except *M7* have the condition of *time spent with Dyskinesias as normal*. *M3* and *L4* have the same feature rules except for Gait. *L2* is a strongly late onset subtype with mostly normal symptoms except for Gait and/or rest tremor amplitude.

### 2.4 Subtype characterization based on PD diagnostic check sheet

Here, we study the six novel subtypes that we discovered in terms of the “Diagnostic Check Sheet” feature category of the LRRK2 dataset (shown in Table 3). The Diagnostic Check Sheet is a collection of assessments used for diagnosing PD in clinical routine. We only keep features from the Diagnostic Check Sheet with less than 30% missing data. We do not observe any patients with the label “Not Applicable”; thus, all values are binary (1 = Yes, 0 = No). We observe that bradykinesia, rigidity, and progressive disorder are present in major proportions (>75%) for all six subtypes, and this proportion is also consistent with the complete LRRK2 dataset. Therefore, it may be considered a characteristic of the LRRK2 dataset rather than something specific to our detected six subtypes. Resting Tremor and “If resting tremor is it 4–6Hx” features are present majorly (>75%) for all subtypes except the *E4* subtype. The frequent presence of these features is expected since bradykinesia, rigidity, and resting tremor are the core motor symptoms upon which patients are diagnosed with PD. The LRRK2 dataset contains a large number of idiopathic PD patients. Thus, the other symptoms are rarely present both in our identified subtypes as well as in the complete LRRK2 data, including *MPTP Exposure, Babinski Sign, Severe levodopa-induced chorea, Neuroleptic treatment at the onset of symptoms, history repeated strokes with the stepwise progression of Parkinsonian features, Sustained remission, Oculogyric crisis, Supranuclear gaze palsy or slowing of vertical saccades, Cerebellar signs, History of definite encephalitis, History of strictly unilateral features after three years*. We also observe that the “Persistent asymmetry affecting the side of onset most” feature is present in >75% for *M3* and *M7* subtypes; between 50 and 75% for the rest of the subtypes. From Table 3, we observe that it is not possible to distinguish the subtypes based on the diagnostic checklist alone in the LRRK2 dataset, highlighting the need for proposed subtype rules based on MDS-UPDRS features. The diagnostic checklist is binary and does not indicate the severity of symptoms. The MDS-UPDRS rating scale is more granular and contains information on the severity of symptoms.

Furthermore, we observe that the *Persistent asymmetry affecting the side of onset most* (DCASYMM) is the most relevant feature to describe these subtypes, as they differ significantly from the characteristics of the overall LRRK2 PD population (shown under the “Overall” column) for five out of the six final subtypes. Both the features of *History of clinical course of 10 years or more* (DCPD10Y) and *History or present at visit with postural instability not caused by other dysfunction* (DCPOSINS) uniquely differentiate three out of the six final subtypes. The highlighted rows (with light green color) in Table 3 are clinical features that differ greatly from the distribution of the complete LRRK2 dataset—*DCASYMM, DCPD10Y, and DCPOSINS*. The clinical features (DCASYMM, DCPD10Y, and DCPOSINS) may indicate the most important diagnostic markers for our six detected subtypes. We observed instances of feature overexpression (higher than the overall population mean) and underexpression (lower than the overall population mean) of the final subtypes when compared with overall population statistics. Furthermore, the *M7* subtype consistently exhibits significantly higher feature values than the overall population means (overexpressed) in terms of our most important diagnostic markers (DCASYMM, DCPD10Y, and DCPOSINS). In contrast, the *L1* subtype shows the opposite trend, consistently underexpressing these markers. However, subtypes L4 and E4 exhibit similar characteristics to the overall population, indicating the limited effect of our identified diagnostic markers.

### 2.5 Subtype characterization based on non-motor symptoms of MDS-UPDRS rating scale

**Figure 4:**
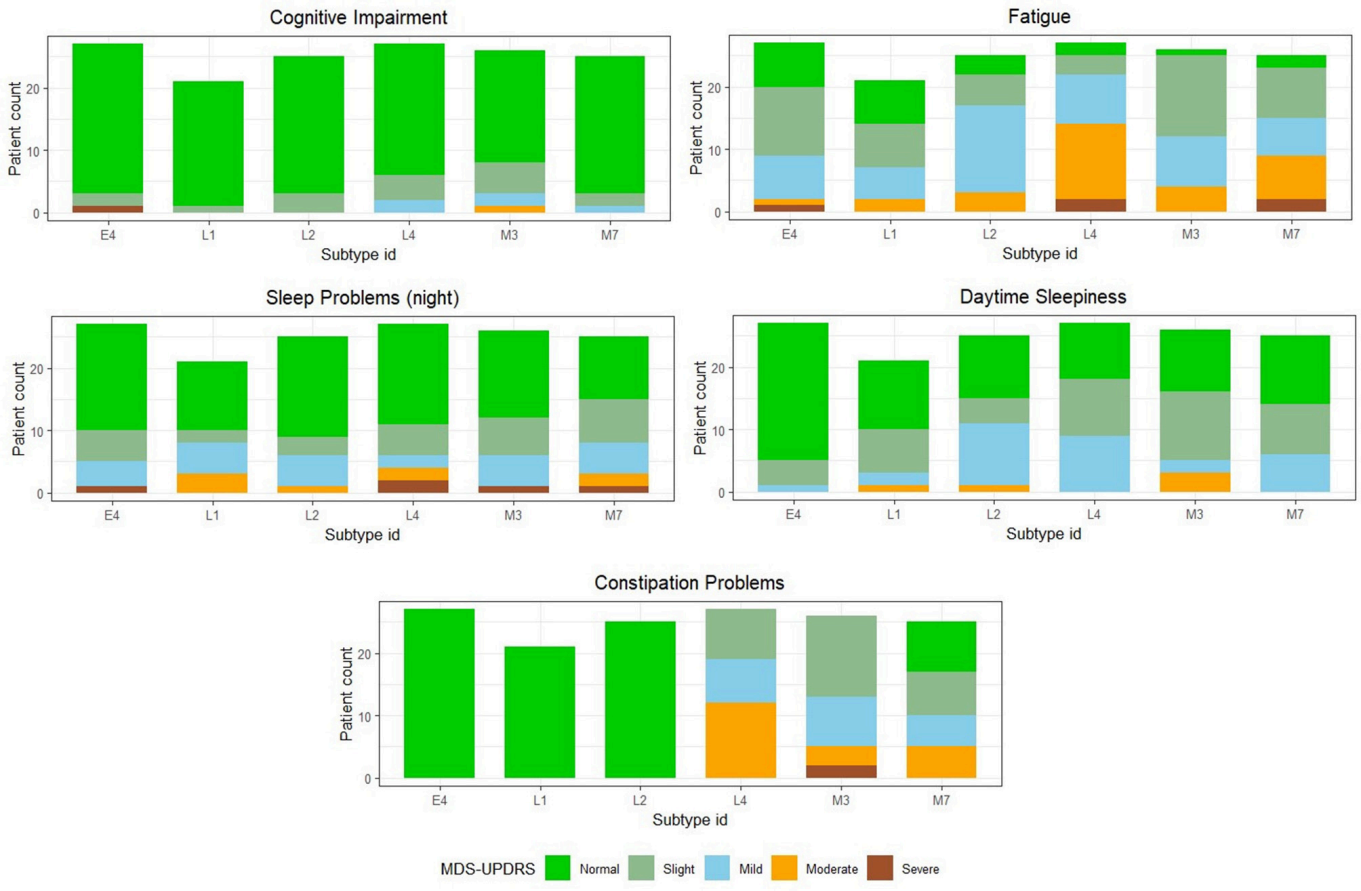
Comparison among the final identified novel subtypes in terms of non-motor features present in the MDS-UPDRS scale where the features follow an ordinal rating scale. Subtype IDs starting with the characters “E,” “M,” and “L” refer to primarily early onset, mixed onset, and primarily late onset PD subtypes, respectively.

**Figure 5:**
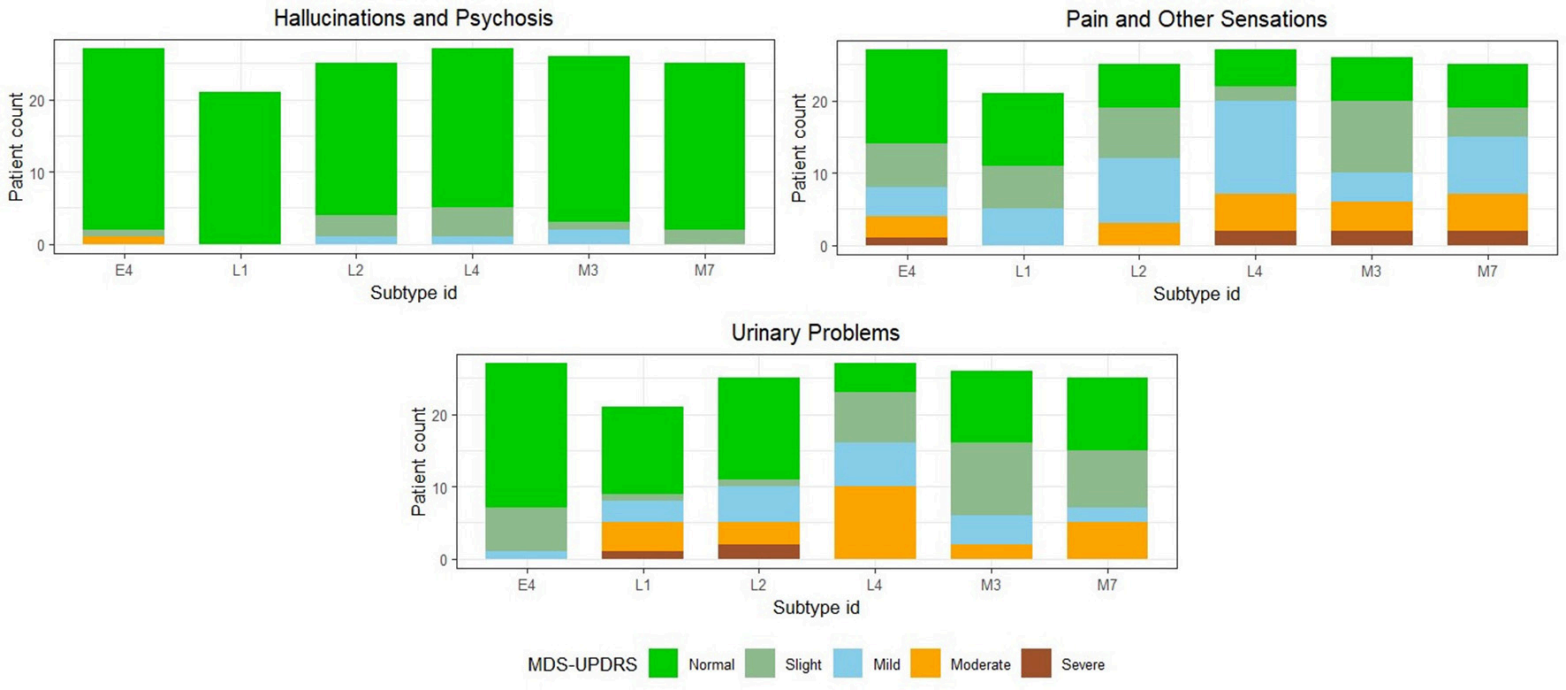
Comparison among the final identified novel subtypes in terms of non-motor features present in the MDS-UPDRS scale, where the features follow an ordinal rating scale. Subtype IDs starting with the characters “E,” “M,” and “L” refer to primarily early onset, mixed onset, and primarily late onset PD subtypes, respectively.

We observe that the majority of features considered for developing the PD classification model, as well as the subtype rules, primarily consist of features from the MDS-UPDRS rating scale. However, certain non-motor features that are shown to be important clinical prognostic features of PD progression, such as mild cognitive impairment, dysautonomia, and REM sleep behavior disorder (RBD), are under-represented in the MDS-UPDRS scale (Fereshtehnejad et al., 2015). Therefore, we further characterize the six novel subtypes in terms of *Part 1: Non-Motor Aspects of Experiences of Daily Living* of the MDS-UPDRS rating scale. We show the subtype comparison in Figures 4, 5.

We observe that *Cognitive impairment, Hallucinations and Psychosis* are almost absent, whereas *Fatigue, Pain and Other Sensations* are consistently present in high proportion across all six subtypes. All subtypes mostly have *Normal* or *Slight* rating scores in case of *Daytime Sleepiness*. For constipation, the major trend is that E4, L1, and L2 are normal due to the simple reason that constipation is included as part of the subtype rules. E4 differs majorly from L4 and M7 in terms of *Urinary Problems* and *Constipation Problems*, where most E4 patients have Normal scores, but M7 and L4 have mostly Mild and Moderate scores. This may be because of two reasons: (i) The E4 subtype has a lower mean PD duration of 6.81 years as compared to M7 and L4, which have 11.6 and 8.89 years, respectively. (ii) The mean assessment age of E4 patients is 49.44 years and it is significantly lower than M7 and L4 which have 63.04 and 66.78 years respectively. Comparing the three primarily late onset subtypes (L1, L2, and L4), L1 is characterized by lower scores on the Geriatric Depression Scale, while patients belonging to L2 show less physical symptoms, such as rigidity and tremor. L4 exhibits a broader range of PD duration and severe fatigue and constipation symptoms, compared to L1 and L2.

### 2.6 Comparative analysis with clustering algorithms and external validation dataset

We compare the demographic characteristics of LRRK2 subtypes with the mapped subtypes of the MDS dataset as well as the clustering algorithms in Table 4.

**Table 4:** Comparison of proposed subtypes (LRRK2 and MDS) and similar clusters obtained from standard clustering algorithms such as HDBSCAN (HDB) and Affinity Propagation (AP).

**Subtype**	**Dataset**	**Patient count**	**Assessment age**	**Age of PD onset**	**Gender (male-female)**	**PD duration**	**Ratio of subjects with LRRK2 mutation**
Comparison with clustering algorithms							
E4	LRRK2	27	49.44 ± 10.46	42.63 ± 10.48	13–14	6.81	0.333
E4	MDS	20	52.40 ± 4.56	44.35 ± 3.87	12–8	8.05	–
HDB9	LRRK2	85	61.46 ± 12.57	56.29 ± 11.7	50–35	5.16	0.435
AP7	LRRK2	100	60.84 ± 12.29	54.95 ± 11.93	58–42	5.89	0.46
M3	LRRK2	26	60.5 ± 10.38	52.77 ± 12.17	17–9	7.73	0.462
M3	MDS	47	66.98 ± 9.04	60.13 ± 10.00	26–21	6.85	–
HDB6	LRRK2	40	61.63 ± 11.65	53.78 ± 12.4	23–17	7.85	0.275
AP6	LRRK2	49	60.69 ± 12.13	52.67 ± 13.27	29–20	8.02	0.306
L4	LRRK2	27	66.78 ± 9.8	57.89 ± 10.94	15–12	8.89	0.481
L4	MDS	25	73.88 ± 9.1	66.56 ± 8.31	17–8	7.32	–
AP4	LRRK2	47	63 ± 10.23	51.74 ± 12.70	25–22	11.26	0.511
Comparison with mapped MDS subtypes							
M7	LRRK2	25	63.04 ± 10.74	51.44 ± 13.29	18–7	11.6	0.24
M7	MDS	38	66.34 ± 8.49	53.50 ± 9.98	22–16	12.84	–
L1	LRRK2	21	65.29 ± 9.37	60.86 ± 8.58	13–8	4.43	0.619
L1	MDS	39	69.05 ± 7.72	63.28 ± 7.38	24–15	5.77	–
L2	LRRK2	25	64.4 ± 9.98	58.6 ± 10.28	11–14	5.8	0.36
L2	MDS	29	75.24 ± 9.78	67.17 ± 10.26	19–10	8.07	–
Overall	LRRK2	397	62.53 ± 12.06	54.50 ± 12.97	214–183	8.03	0.403
Overall	MDS	402	67.42 ± 9.96	59.23 ± 10.67	250–152	8.2	–

Subtype IDs starting with the characters “E,” “M,” and “L” refer to primarily early onset, mixed onset, and primarily late onset PD subtypes, respectively. LRRK2 mutation information is not available for the MDS dataset.

#### 2.6.1 Comparison with mapped subtypes from the MDS dataset

We observe that the MDS patients are older on average (67.42 vs. 62.53 years of age) and have a delayed PD onset (59.23 vs. 54.50 years of age), as compared to the LRRK2 patients; this was also reflected in the demographic characteristics for all the subtypes. The PD duration was quite consistent between the LRRK2 and MDS subtypes with an average difference of 1.42 years; subtype L2 had the maximum difference of 2.27 years. We observed that Subtype L2 and L4 in MDS had the most amount of differences than its LRRK2 counterpart, in terms of age of PD onset; PD onset in MDS was delayed by 8.62 years on average.

#### 2.6.2 Comparison with clustering algorithms

Clustering approaches are known to be highly sensitive to changes, where differences in features and datasets have a vast impact on the resulting subtypes. This limits the comparability of subtypes derived from such methods by other authors. Therefore, we performed our own clustering with the same setup as with the decision tree. We compare the subtypes obtained by our proposed decision tree-based PD subtyping method with two popular clustering algorithms, such as Affinity Propagation (Frey and Dueck, 2007) and HDBSCAN (Campello et al., 2013). We used the “AffinityPropagation” function of the *scikit-learn* package (sklearn.cluster) and “HDBSCAN” from the *hdbscan* package, utilizing their default hyperparameter values. We impose the same criteria for the minimum size of a subtype to be 10, and thus remove clusters with fewer than 10 patients.

HDBSCAN and affinity propagation clustering algorithms form eight and seven clusters, respectively, which is quite close to our proposed number of novel subtypes (clusters), which is six. We use Rand's Index (Rand, 1971) to compute the consistency among the clustering assignments. We observe a high similarity score between *Affinity Propagation* and *HDBSCAN* with Rand's Index of 0.926. Compared to our proposed novel subtypes, we observe moderate similarity values in terms of Rand's Index, being 0.588 and 0.591 when comparing our proposed novel subtypes with Affinity Propagation and HDBSCAN clustering algorithms, respectively. This highlights the difference between unsupervised clustering-based methods and age-of-onset-guided methods, such as the decision tree used.

**Figure 6:**
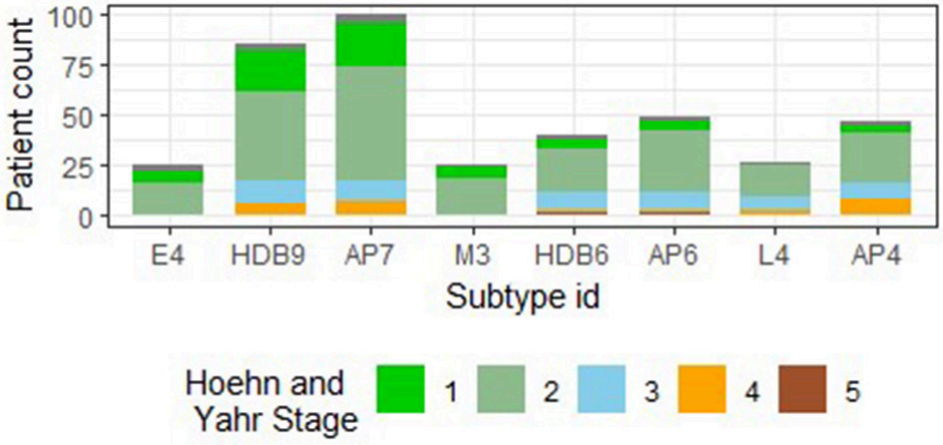
Comparison of proposed subtypes and similar clusters obtained from standard clustering algorithms such as HDBSCAN (HDB) and Affinity Propagation (AP), in terms of Hoehn and Yahr Scale (NHY) values. Subtype IDs starting with the characters “E,” “M,” and “L” refer to primarily early onset, mixed onset, and primarily late onset PD subtypes, respectively.

As Rand's Index provides only an aggregate similarity statistic between clustering results and our proposed subtypes, we investigate at an individual cluster level. We select clusters obtained from HDBSCAN and Affinity Propagation clustering algorithms that overlap with at least 10 patients with our proposed subtypes. In Table 4, we observe a similarity in terms of PD duration and percentage of subjects with LRRK2 mutation. Figure 6 also compares the Hoehn and Yahr Scale (NHY) values. We observe that: (i) Our E4 subtype overlaps quite well with cluster 9 of the HDBSCAN clustering algorithm, referred to as the “HDB9” cluster, and cluster 7 of the Affinity Propagation clustering algorithm, referred to as “AP7.” (ii) Our M3 and L4 subtype shows good overlap with HDB6 and AP6 clusters, having 10 and 12 patients, respectively. (iii) Our L4 subtype also shows good overlap with the AP4 cluster, with 12 patients, respectively.

## 3 Discussion

Parkinson's Disease is highly heterogeneous both in clinical and pathobiological mechanisms (Von Coelln et al., 2021). An improved understanding of different subtypes of Parkinson's Disease may facilitate better diagnostics and the development of new therapeutic approaches. Hypothesis-driven subtyping mainly focusses on motor symptoms, while data-driven subtyping often remains challenging to understand and interpret (Deng et al., 2024). In general, the subtyping of PD faces two distinct challenges: methodological quality and clinical applicability (Mestre et al., 2021). Common methodological shortcomings in previous studies of PD subtyping included the underlying study population not being representative of PD patients (i.e., single-center recruitment) and the data used for subtyping often being limited to one clinical domain, rather than comprehensive. Additionally, the temporal stability of subtypes during disease progression is seldom assessed, and internal or external validation is rarely performed (Mestre et al., 2021). Two newer data-driven studies (Su et al., 2024; Hähnel et al., 2024) focus on progression and progression pace subtypes. Hähnel et al. (2024) found two distinct subtypes of PD progression that are stable across cohorts and align with the brain-first vs. body-first concept. Su et al. (2024), on the other hand, discovered three pace oriented subtypes (inching, moderate, rapid). They suggest neuroinflammation, oxidative stress, metabolism, PI3K/AKT, and angiogenesis pathways as potential drivers for rapid PD progression. Further, they identified *STAT3, FYN, BECN1, APOA1, NEDD4*, and *GATA2* as potential driver genes for the rapid pace subtype. Park et al. (2025) recently suggested using wearable sensors attached bilaterally to body segments to classify PD severity subtypes and track disease progression. Regarding the temporal stability of PD subtypes, other studies have found that tremor-dominated phenotypes likely transition to a postural-instability-gait-disorder-dominated phenotype with disease progression (Von Coelln et al., 2021; Simuni et al., 2016). In this work, the LRRK2 and MDS datasets are cross-sectional in nature and do not contain any longitudinal data of the participants, and thus, we could not compare prognosis between the proposed novel subtypes. We identified robust subtypes in two independent cohorts from different continents. The selected patients of the LRRK2 dataset are recruited from the *National Institute of Neurology* in Tunis, Tunisia. This cohort exhibits some peculiarities compared to other LRRK2 and more general PD cohorts. At the genetic level, North African Berber patients are known to have higher mutation frequencies in the LRRK2 gene, with homozygous G2019S mutations being particularly frequent (Lesage et al., 2006; Trinh et al., 2014). On the phenotype level, lower years of education (Sassi et al., 2012), which is known to negatively influence frontal assessment battery (FAB) scores (Beato et al., 2007), must be highlighted. Regarding the age of disease onset, lifestyle factors have been shown to have an effect in a Tunisian cohort (Lüth et al., 2020). Due to these peculiarities, the found subtypes exhibit the risk of being LRRK2 or cohort-specific. An argument against this is the ratio of LRRK2 mutation carriers of the subtypes. For all subtypes except *Subtype L1*, the ratio is close to the population's overall ratio of 30%–40% mutation carriers (Hulihan et al., 2008; Lesage et al., 2005, 2006). The MDS dataset comprises patients from the UK and the US, representing multiple sites. The evaluation of the subtypes found in the LRRK2 dataset in an independent cohort (MDS) ensures generalizability and reproducibility between cohorts, thereby overcoming these challenges. Still, due to our experimental setup, additional subtypes may exist, and we do not claim completeness of the subtypes found. One future direction is to include Asian cohorts, who typically exhibit lower to no G2019S mutation rates (Tan et al., 2005). We expect that by broadening the cohort, more subtypes will be found, potentially with a focus on non-motor symptoms, as these are underrepresented in the LRRK2 dataset. Furthermore, we anticipate that subtypes with minor variations compared to our subtypes will be identified. While being more robust against changes in the dataset compared to clustering approaches, there may still be further features that describe our subtypes and were not identifiable here, as they are absent in our study. Due to the evaluation in an independent cohort, we are confident that our subtypes are robust.

By using the *MDS-UPDRS rating scale*, which encompasses both motor and non-motor symptoms, and the *Geriatric Depression Scale*, the subtyping utilized comprehensive data sources that can reflect the complex phenotypes observed in PD. Regarding the *Geriatric Depression Scale*, a critical point is the usage in non-geriatric cohorts such as EOPD patients, as it was designed explicitly for geriatric cohorts. It can be argued that EOPD patients are closer to geriatric cohorts in terms of fragility and motor limitations than to same-age healthy cohorts. Therefore, the *Geriatric Depression Scale* feature could be used. In a potential diagnostic use of the subtyping rules, the Geriatric Depression Scale is not applicable and could potentially be replaced, after evaluation, with an alternative depression assessment for general cohorts. In our study, we relied on semi-publicly available data and did not influence the assessments used. Furthermore, the focus on these standardized scales ensures the easy applicability of our subtyping rules to future cohorts and individual patients. Nonetheless, a significant effort was necessary to map the features between the two data sources. This highlights one well-known limitation of subtyping approaches for PD: a standardized reporting scheme is missing (Qian and Huang, 2019). Fereshtehnejad et al. (2015) observed that MDS-UPDRS is inferior to composite outcomes with an equally weighted impact of important non-motor features such as cognitive impairment and REM sleep behavior disorder (RBD) to predict major outcomes such as dementia or more rapid disease progression. In other words, using only MDS-UPDRS features for subtyping, the importance of some non-motor features, such as MCI, dysautonomia, and RBD, is under-represented in the final solution, all of which are essential clinical prognostic features of PD progression. This introduces the risk of bias toward motor features, which is a limitation of our work. Still, Lin et al. (2025) were able to show significant differences in brain regions, including the posterior cingulate gyrus, lenticular nucleus, olfactory cortex, and cerebellum, for subtypes mostly based on motor symptoms they found by clustering. We assume that using a dataset enriched with additional non-motor symptoms would enhance the subtyping rules identified using motor symptoms to a greater extent. Nonetheless, our evaluation in an independent cohort demonstrated that, despite this limitation, the identified subtypes are valid beyond the North African Berber community. Furthermore, as described previously, we assume that additional subtypes exist that may be uncovered by including more features in the analysis. In summary, our approach overcomes most of the typical methodological limitations of similar subtyping studies.

While most methodological issues of PD subtyping can be addressed by following expert recommendations and mindful study design, the clinical applicability of subtypes in PD is a challenge of a different magnitude. The clinical applicability of subtypes is determined by the ease of use in clinical daily routine and the benefits derived from subtyping patients (Mestre et al., 2021). Our data-driven decision tree-based approach overcomes the issue of reproducibility of classical clustering-based approaches (Qian and Huang, 2019). This study defines simple subtyping rules based on typical clinical assessments, enabling fast, inexpensive, and low-tech subtyping in the clinical routine. The clinical benefits of PD subtyping in past studies were assessed to be questionable by Mestre et al. (2021) in a comprehensive analysis of PD subtyping studies. Still, validation in additional datasets would further strengthen the proposed subtyping rules. To provide prognostic value or treatment implications, subtypes should be characterized biologically by identifying specific biomarkers. By applying our subtype rules to other PD cohorts, which are rich in molecular data, we will be able to explore these aspects in the future. Instead of using unimodal data as used by Markello et al. (2021). An essential next step is to find genetic markers for the subtypes (Qian and Huang, 2019). Here, the LRRK2 genetic data could be utilized to identify small nuclear polymorphisms that characterize the subtypes. Further clinical validation studies, particularly with regard to the prognostic value and potential treatment implications, are necessary to reach a definitive conclusion regarding the clinical benefits of our subtyping approach. In terms of clinical applicability, the six subtypes identified in our study may provide relevant guidance for prognosis and therapeutic strategies. The primarily early onset subtype E4 (Table 4, Figure 3) was characterized by preserved gait, low non-motor symptom burden, and shorter disease duration, which may indicate a comparatively favorable prognosis. However, early onset patients are known to be more prone to treatment-induced motor complications over time (Schrag and Schott, 2006). The mixed onset subtypes M3 and M7 (Tables 4, 5, Figure 3) both showed high rates of persistent asymmetry and non-motor symptoms, including constipation and depression (M3), as well as postural instability and longer disease duration (M7). These constellations may predict faster functional decline and suggest that patients in these groups could benefit from intensified supportive measures such as physiotherapy, fall-prevention programs, and early screening for affective disorders. Within the late onset group, **L1** displayed low depression scores and relatively mild motor involvement, potentially reflecting a more benign course with implications for patient counseling and monitoring intensity. In contrast, L2 and L4 were associated with more pronounced gait impairment, tremor, and, in the case of L4, severe fatigue and autonomic symptoms (Figure 5), all of which are established predictors of reduced quality of life and increased care dependency (Chaudhuri et al., 2006). Taken together, these clinically distinct profiles illustrate how data-driven subtyping could inform patient stratification, prognosis estimation, and prioritization of non-pharmacological interventions, while also providing a framework for designing subtype-specific clinical trials. The clinical relevance of disease subtyping in other medical disciplines has been a topic of controversy until its importance for therapeutic decision-making was demonstrated in clinical trials.

**Table 5:** Feature mapping between LRRK2 and MDS datasets, which are the training and external validation dataset respectively.

**LRRK2 feature name**	**Label**	**MDS feature name**	**LRRK2 feature name**	**Label**	**MDS feature name**
Exact matches					
NP1COG	Cognitive impairment	mdsupdrs1_1	NP3HMOVR	Hand movements—Right hand	mdsupdrs3_5a
NP1HALL	Hallucinations and psychosis	mdsupdrs1_2	NP3HMOVL	Hand movements—Left hand	mdsupdrs3_5b
NP1DPRS	Depressed moods	mdsupdrs1_3	NP3PRSPR	Pronaton-supination—Right hand	mdsupdrs3_6a
NP1ANXS	Anxious mood	mdsupdrs1_4	NP3PRSPL	Pronaton-supination—Left hand	mdsupdrs3_6b
NP1APAT	Apathy	mdsupdrs1_5	NP3TTAPR	Toe tapping—Right foot	mdsupdrs3_7a
NP1DDS	Features of dopamine dysregulation syndrome	mdsupdrs1_6	NP3TTAPL	Toe tapping—Left foot	mdsupdrs3_7b
NP1SLPN	Sleep problems (night)	mdsupdrs1_7	NP3LGAGR	Leg agility—Right leg	mdsupdrs3_8a
NP1SLPD	Daytime sleepiness	mdsupdrs1_8	NP3LGAGL	Leg agility—Left leg	mdsupdrs3_8b
NP1PAIN	Pain and other sensations	mdsupdrs1_9	NP3RISNG	Arising from chair	mdsupdrs3_9
NP1URN	Urinary problems	mdsupdrs1_10	NP3GAIT	Gait	mdsupdrs3_10
NP1CNST	Constipation problems	mdsupdrs1_11	NP3FRZGT	Freezing of gait	mdsupdrs3_11
NP1LTHD	Lightheadedness on standing	mdsupdrs1_12	NP3PSTBL	Postural stability	mdsupdrs3_12
NP1FATG	Fatigue	mdsupdrs1_13	NP3POSTR	Posture	mdsupdrs3_13
NP2SPCH	Speech	mdsupdrs2_1	NP3BRADY	Global spontaneity of movement	mdsupdrs3_14
NP2SALV	Saliva and drooling	mdsupdrs2_2	NP3PTRMR	Postural tremor—Right hand	mdsupdrs3_15a
NP2SWAL	Chewing and swallowing	mdsupdrs2_3	NP3PTRML	Postural tremor—Left hand	mdsupdrs3_15b
NP2EAT	Eating tasks	mdsupdrs2_4	NP3KTRMR	Kinetic tremor—Right Hand	mdsupdrs3_16a
NP2DRES	Dressing	mdsupdrs2_5	NP3KTRML	Kinetic tremor—Left hand	mdsupdrs3_16b
NP2HYGN	Hygiene	mdsupdrs2_6	NP3RTARU	Rest tremor amplitude—RUE	mdsupdrs3_17a
NP2HWRT	Handwriting	mdsupdrs2_7	NP3RTALU	Rest tremor amplitude- LUE	mdsupdrs3_17b
NP2HOBB	Doing hobbies and other activities	mdsupdrs2_8	NP3RTARL	Rest tremor amplitude- RLE	mdsupdrs3_17c
NP2TURN	Turning in bed	mdsupdrs2_9	NP3RTALL	Rest tremor amplitude- LLE	mdsupdrs3_17d
NP2TRMR	Tremor	mdsupdrs2_10	NP3RTALJ	Rest tremor amplitude- lip/jaw	mdsupdrs3_17e
NP2RISE	Getting out of bed, car, deep chair	mdsupdrs2_11	NP3RTCON	Constancy of rest	mdsupdrs3_18
NP2WALK	Walking and balance	mdsupdrs2_12	DYSKPRES	Were dyskinesias present	mdsupdrs_dysk
NP2FREZ	Freezing	mdsupdrs2_13	DYSKIRAT	Did movements interfere with rating	mdsupdrs_int
PDMEDYN	On Meds to treat PD symptoms	mdsupdrs3a	NHY	Hoehn and Yahr Stage	mdsupdrs_hy
PDCLNSTA	If on Meds, what is clinical state	mdsupdrs3b	NP4WDYSK	Time spent with dyskinesias	mdsupdrs4_1
LDOPARX	On levodopa	mdsupdrs3c	NP4DYSKI	Functional impact of dyskinesias	mdsupdrs4_2
MINSNCLD	If on levodopa, minutes since last levodopa dose	mdsupdrs3c1	NP4OFF	Time spent in OFF state	mdsupdrs4_3
NP3SPCH	Speech	mdsupdrs3_1	NP4FLCTI	Functional impact of fluctuations	mdsupdrs4_4
NP3FACXP	Facial expression	mdsupdrs3_2	NP4FLCTX	Complexity of Motor Fluctuations	mdsupdrs4_5
NP3RIGN	Rigidity—Neck	mdsupdrs3_3a	NP4DYSTN	Painful OFF-state Dystonia	mdsupdrs4_6
NP3RIGRU	Rigidity—RUE	mdsupdrs3_3b	NHY	Hoehn and Yahr stage	hy
NP3RIGLU	Rigidity—LUE	mdsupdrs3_3c	MCATOT	MoCA total score	moca
NP3RIGRL	Rigidity—RLE	mdsupdrs3_3d	Gender	Gender	sex
NP3RIGLL	Rigidity—LLE	mdsupdrs3_3e	Ageonset	Age at onset	pdonset
NP3FTAPR	Finger tapping right hand	mdsupdrs3_4a	totled	Total levodopa equivalent dose	ldopadose_LED
NP3FTAPL	Finger tapping left hand	mdsupdrs3_4b			
Indirect matches (reviewed by medical doctor)					
demopd_ageassess	Age in years at assessment date (DEMOPD CRF)	Age			
demopd_ageassess minus ageonset	PD duration in years	durat_pd			
epworth_sum	Epworth sleepiness scale	Product of *mdsnms_K3f* and *mdsnms_K3s*			

As mentioned, subtyping by clustering is highly sensitive, leading to low inter- and intra-dataset comparability. Therefore, we compared the decision tree-based results with the clustering-based results, using the same preprocessing and filtering steps. Nonetheless, we would like to include a shallow comparison to Lawton et al. (2018). Similarities in the progression speed, mildness, and potentially physicality can be assumed. The subtypes M3 and M7 from our study share similarities with the fast motor-progression subtype, the first subtype from Lawton et al., with the difference that asymmetrical symptoms characterize our subtypes, while *Lawtons* are symmetrical-symptom-based. E4 is comparable with *Lawtons* second subtype. Both are characterized by comparably mild symptoms and an early onset. Further, *Lawtons* third subtype, which is characterized by severe physical and psychological states, shows some similarities with L4. Still, L4 also appears to encompass a comparably mild spectrum. However, we must note again that too much weight should not be given to this comparison.

To date, no subtyping approach for PD has translated into clinical validation. Fereshtehnejad et al. (2017) have worked toward making clinical adoption easier; however, it has not yet been translated into clinical practice. Few PD subtyping studies have also performed external validation of their subtypes in independent cohorts. Lawton et al. (2018) employ a clustering-based approach to identify four PD subtypes, which are characterized by motor, cognitive, and non-motor symptoms. The patients in the analyzed *Tracking Parkinson's and Discovery* cohort were assessed within 3.5 years of diagnosis and followed up longitudinally. The proposed subtypes were defined mainly by disease progression and symptom severity. As the used cohorts in this study did not have information on disease progression, a direct comparison is not possible. Furthermore, all four proposed subtypes in Lawton et al. (2018) consisted of a mixture of patients with early onset and late onset PD. Our more granular subtypes, with distinctions in age of onset, can provide better insight into the differences between these patient groups. van Rooden et al. (2011) also used a clustering-based analysis to identify subtypes of motor and non-motor features of PD. For training, a Dutch cohort comprising 344 patients was utilized, while the identified four subtypes were evaluated using longitudinal data from both the same cohort and an independent Spanish cohort with 357 patients. The found subtypes mainly differ in the severity of non-dopaminergic features and motor complications. They found two EOPD and two LOPD clusters. Both age-of-onset-based groups differed in terms of severity. Their first cluster, which consists of young patients with mild severity, appears to be similar to our *Early 4* subtype; however, since neither explicit feature values nor rules for the subtypes are provided, a direct match between the results is not possible.

We observe that only six out of 17 candidate subtypes satisfy our filtering criteria in Table 1. The reasons for failing on external validation data may be as follows: (i) mismatch between patient population demographics (as highlighted in the above point: LRRK2 final subset—Tunisia, whereas MDS data—US and UK). It may result in differences in culture, lifestyle, and other environmental factors. (ii) mismatch in nature of cohort objective (LRRK2 is mutation-specific, whereas MDS has no such constraint). Each subtype is described by the “AND” operation of multiple feature conditions, i.e., every condition must be satisfied to assign a given patient to a particular subgroup. The order of feature conditions in Figure 2 does not indicate a feature's importance for determining a patient subtype. A better approach is to examine the unique feature conditions that distinguish the given subtype from the others. Comparing two clustering-based approaches with our decision tree-based approach, we found a high concordance of subtypes between the two clustering-based approaches and a slight concordance between the decision tree and each of the clustering-based approaches. A possible reason is the additional information that the age-of-onset introduces, shifting the results more in this direction. Still, we expect the age-of-onset-based approach's subtypes to be more robust compared to clustering-based approaches in terms of the cohort's composition.

The next steps include investigating genetic differences in the six valid subtypes. Here, the LRRK2 single-nucleotide polymorphisms (SNPs) data can be used to identify mutations characterizing the subtypes. If identified, significant SNPs require validation in a separate cohort or in an *in vitro* experiment. Furthermore, we plan to validate our results on the PPMI cohort and to investigate other machine-learning approaches that examine unique feature conditions for distinguishing between the subtypes.

## 4 Methods

**Figure 7:**
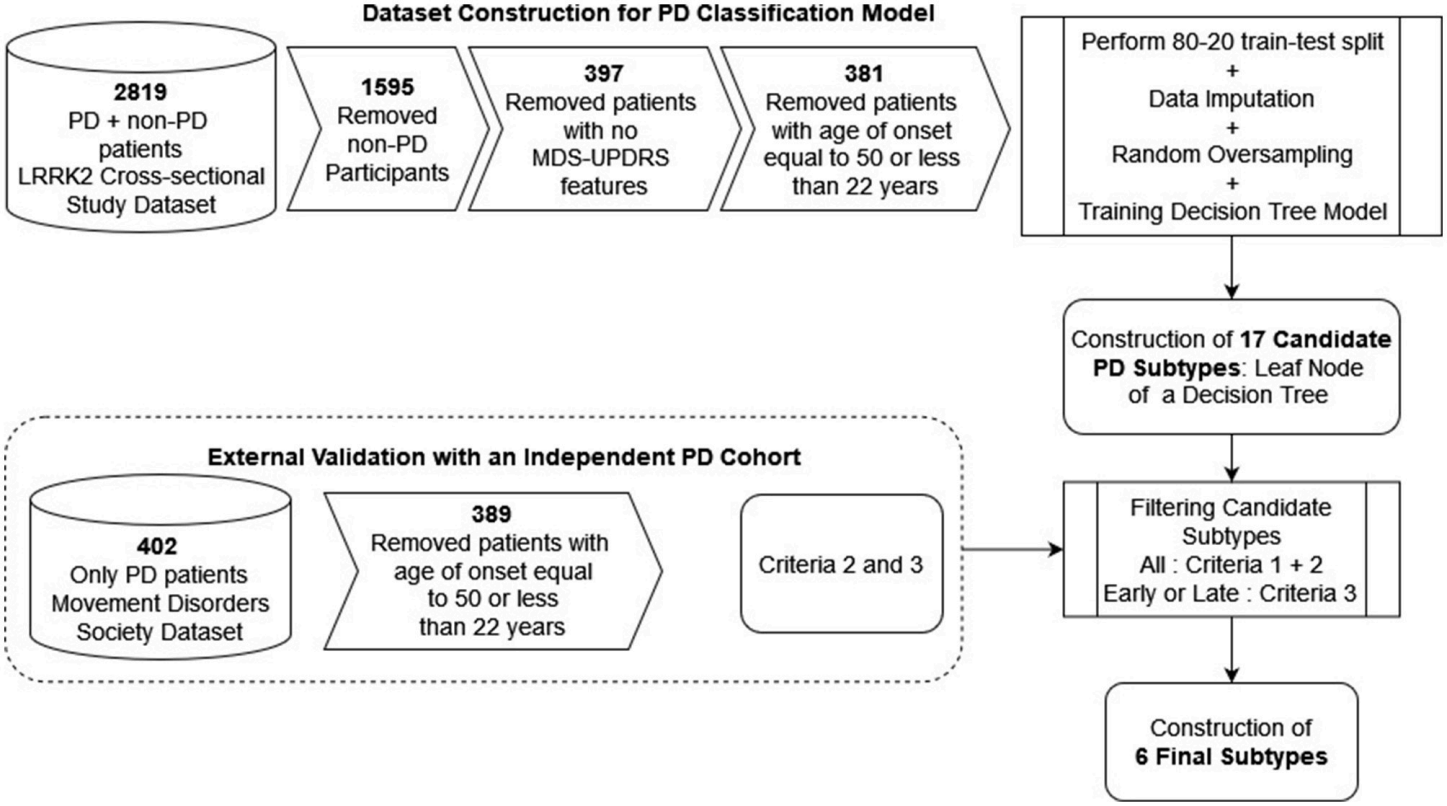
Method workflow for training the decision tree-based PD classification model and obtaining the final PD subtypes.

We explain the complete workflow involved in developing the PD classification model and subsequently identifying the six final PD subtypes in Figure 7.

### 4.1 Study population

The LRRK2 Cohort Consortium (LCC) was established to assemble and study groups of individuals with and without Parkinson's disease who carry mutations in the LRRK2 gene. It comprises three closed studies: the *LRRK2 Cross-sectional Study, LRRK2 Longitudinal Study*, and the *23andMe Blood Collection Study*. The LCC followed standardized data acquisition protocols, and clinical data and biological samples are stored in a comprehensive Parkinson's database and biorepository, respectively (Michael J. Fox foundation for Parkinson's Research, 2023). Complete clinical data of the patients were obtained from the “LRRK2 Cross-sectional Study” of the LRRK Cohort Consortium. Further exclusion is based on three criteria, which are described in detail below: (1) study participants without PD, (2) complete absence of values in features that are necessary for mapping between data sets, and (3) age-of-onset below 22 years or equal to 50 years.

We only consider the 2017 study patients with PD, which equals 1595 patients. Since we also perform a validation study on an independent PD cohort (MDS), we select only those features that overlap between the LRRK2 and MDS datasets. Seventy-one out of 80 (88.75%) overlapping features belong to the MDS-UPDRS rating scale. Table 5 shows the mapping at the level of individual features between LRRK2 and MDS datasets. Therefore, we remove patients whose values for the MDS-UPDRS rating scale are entirely missing, resulting in 397 patients from the LRRK2 dataset for this study. We observe that all the 397 patients are from the National Institute of Neurology (Tunis, Tunisia) and belong to the “Arab-Berber” race with the average age of PD onset of 54.50 ± 12.97 years. Particularities arising from this study population are covered in the discussion section. The MDS dataset's study population comprises patients with PD from the United Kingdom and the United States of America. There is no sequencing or longitudinal data available for these patients in the LRRK2 dataset, and therefore, we limit our analysis to only cross-sectional clinical data. Therefore, we cannot analyze the prognosis of the novel subtypes. 40.3% of 397 patients carry the LRRK2 G2019S mutation, which is comparable to the natural occurrence of this mutation in about 32.96% of the sampled population in Tunisia (Landoulsi et al., 2017). Simpson et al. (2022) observe a high degree of heterogeneity in the estimates of the prevalence of LRRK2 variants across different ethno-racial groups; G2019S occurs more frequently in Ashkenazi Jews and North African Berber Arabs (Lesage et al., 2008; Ishihara et al., 2007), and rarely in East Asian populations.

### 4.2 Data preparation

Given the reduced patient subset of LRRK2 data of 397 patients with 80 features (the number of features that overlap between LRRK2 and the MDS dataset), we now prepare the data to make it suitable for the PD classification model. We begin by formulating the binary classification task of predicting whether a patient has early- or late onset PD. The classification approach has some advantages over the regression task as a first methodology for investigation. First, it is closer to the current state-of-the-art clustering in that the age of onset is used as a guide to identify novel subtypes (classes/clusters). Second, filtering and supervision are easier in the classification setup, but can still be transferred to a regression setting. The terms “early onset PD” (EOPD) and “young onset PD” (YOPD) have been used interchangeably in the literature. EOPD is defined as the type of PD with an age at onset (AO) of more than 21 years and less than the usual AO for PD; this maximal age used for EOPD has varied between 40 and 60 years across different studies. Mehanna et al. (2022) investigated this exact issue and finally defined EOPD as AO greater than 21 years and less than 50 years. We utilize this formulation and define late onset PD (LOPD) as AO greater than 50 years. We remove patients with an age of PD onset equal to fifty years (boundary of early and late onset PD) or less than 22 years (juvenile-onset PD). Therefore, our final dataset comprises 381 patients from either early or late onset PD. We observe that 40.9% of these 381 patients carry the LRRK2 mutation. For binary classification, the late onset PD patient is assigned a positive label of 1, and the early onset PD patient is assigned a label of 0. The resultant LRRK2 dataset primarily comprises Stage 2 (58.0%) patients, followed by Stage 3 (14.17%), Stage 1 (13.12%), Stage 4 (9.97%), and Stage 5 (2.10%) patients according to the Hoehn and Yahr Scale.

As part of data preprocessing, we perform the following set of feature selection steps: (i) remove features with more than 15% missing data, (ii) remove all age onset-related features like age of assessment (present age), PD duration, and age of PD onset, since our target label (early or late onset PD) is directly computed based on the age at PD onset. (iii) perform a multi-collinearity check through which we remove features that are linearly dependent on each other, such as multiple age-related features like age of taking the different tests and age while filling the questionnaires. Therefore, our final feature set consists of 72 features: (i) 70 features of the MDS-UPDRS rating scale, (ii) the Geriatric Depression Scale (numeric value on a scale of 0 to 15), and (iii) gender. The MDS-UPDRS rating features comprise four parts: *Part 1: non-motor experiences of daily living, Part 2: motor experiences of daily living, Part 3: motor examination*, and *Part 4: motor complications*. The features usually follow an ordinal rating scale of 0 to 4: *Normal, Slight, Mild, Moderate*, and *Severe*. The resultant LRRK2 dataset has few missing values; *Did movements interfere with rating* has the highest missing percentage of 9.45%, followed by *Calculated Total Score for Geriatric Depression Scale-15* and *If on Levodopa, minutes since last levodopa dose* of 5.77 and 2.36% respectively. All the remaining features have less than 0.79% missing values. We perform data imputation to mitigate the missing values using normative values—“median” for numeric and “mode” for categorical (computed using training data only) since this is more interpretable than complex Random Forest-based data imputation methods like *MissForest* (Stekhoven and Bühlmann, 2011).

### 4.3 PD classification model and construction of candidate PD subtypes

We follow the 80–20 train-test split, which results in 304 and 77 data points for the training and testing datasets, respectively. We observe a significant class imbalance issue, where late onset PD patients outnumber early onset PD patients (197 and 107 patients, respectively). This is consistent with the real-life observation that only 5%−10% of patients diagnosed with PD have early onset PD. Therefore, we perform the random oversampling method, where we resample from the minority class to equal the number of training data points for both classes.

We now train a decision tree binary classification model to distinguish between early- and late onset PD patients. Next, we perform 10-fold cross-validation with *minimum samples per leaf* (10, 12, 14, 16, 18, 20) and *maximum tree depth* (4, 8, 12, 16, 20) features for hyperparameter optimization (*GridSearch* method). We use the *learned decision tree and perform traversal from the root to the leaf with a positive label (patients with PD)* to obtain the patient subtype rules, simply a series of feature conditions connected with *AND* conditions. During hyperparameter tuning, we restrict the *minimum samples per leaf* parameter to be at least 10 to allow sufficiently large subtypes, i.e., subtypes with a minimum of 10 patients. Until now, we consider each leaf of the learned decision as a candidate subtype. We categorize a candidate subtype as *early onset* when most of them are EOPD patients (sufficiently skewed, with Gini impurity ≤ 0.4), *late onset* when most of them are LOPD patients, and *mixed onset* when there is no clear majority between EOPD and LOPD patients (Gini impurity >0.4); Gini impurity ranges from 0 (highly skewed toward one class) to 0.5 (no skewness).

### 4.4 Filtering of candidate PD subtypes to form final subtypes

We now describe the three criteria for filtering the candidate subtypes and obtaining the final (meaningful and novel) subtypes.

Criteria 1. Subtypes obtained should be large enough in the training data (LRRK2 dataset) and should contain at least 10 unique patients. Since we impose this constraint on the learned decision tree, we observe that early onset PD patients will have duplicates due to oversampling and, thus, will not accurately represent the correct number of unique patients. Specifically, the training data with the early onset PD label has an 84.1% chance of being present twice. Thus, the *minimum samples per leaf* for late onset PD is 10 patients, while for early onset PD, it is 19 patients (if 107 patients are unique among 197 patients in the training data, then 10 unique patients are found in 197/107*10 = 18.4).

Criteria 2. To avoid weakly validated subtypes, there should be sufficient positive matches between the same age-onset-based subtype categories of training data (LRRK2 dataset) and external validation data (MDS dataset). We set the threshold (i.e., the minimum number of matched patients) to 5% to prevent overfitting based on the limited cohort and to increase generalizability. Subtypes with less than 5% of associated individuals are not considered significant and are potentially not clinically relevant as they carry the risk of reflecting outliers; van Rooden et al. (2011) utilized the same threshold to discard small-sized clusters in case of PD subtyping. While more permissive thresholds may lead to the identification of additional subtypes, they also increase the risk of overfitting and the inclusion of clinically irrelevant subtypes. This means that the *early onset* candidate subtype of LRRK2 data should match with at least 5% of early onset PD patients of MDS data (5% of 68 = 3.4); the same leads to 17 (5% of 321) patients for a *late onset* candidate subtype. Since a mixed onset candidate subtype has no clear majority between EOPD and LOPD patients, we conclude that such subtypes may be independent of age of onset (i.e., they cannot be defined in terms of age of PD onset). Therefore, we compute the positive match with the entire MDS dataset and keep its threshold as 21 (5% of 402 = 20.1).

Criteria 3. This is an additional criterion for early and late onset PD subtypes where the skewness to early or late onset PD remains consistent between training data (LRRK2 dataset) and external validation data (MDS dataset). For example, for a subtype categorized as *Early onset* in the LRRK2 data, the match percentage in the MDS data should be higher for early onset PD patients compared to late onset PD patients. The precision should be above the threshold of random chance. If we randomly select a patient from (321 late onset + 68 early onset PD): 82.5% probability of being late onset PD, 17.5% probability of being early onset. Thus, a higher chance than random indicates skewness toward a particular age-onset class. Precision for a given subtype, such as the *Early 4* subtype, is defined as the ratio of positive matches with early onset PD patients in the MDS data to the total patient count of the entire MDS dataset (including both early and late onset PD patients). We do not impose this (precision-based) criterion on the *mixed onset* candidate subtypes because it is independent of the age of PD onset and thus precision cannot be reasonably defined in terms of either early or late onset PD.

### 4.5 Characterization study of final subtypes

Till now, we have utilized only overlapping features between LRRK2 and MDS datasets (see Table 5 for the list of features) to train the decision tree classification model and derive the final subtype rules; these features mostly consist of MDS-UPDRS rating scale features. However, for the characterization study, we consider all features of LRRK2 data that have less than 15% missing data. In Figure 3, we visualize the distribution of feature values among the final subtypes in terms of violin plots. Here, an additional feature of interest is the Hoehn and Yahr stage, which measures the degree of progression of PD symptoms in a patient. It is an ordinal scale from 0 to 5 (0: Asymptomatic, 1: Unilateral involvement only, 2: Bilateral involvement without impairment of balance, 3: Mild to moderate involvement, 4: Severe disability, still able to walk or stand unassisted, 5: Wheelchair-bound or bedridden unless aided). We represent the subtype characteristics in terms of the mean and standard deviation of the feature values of all patients in that subtype, as reported in Table 3. The values under *Diagnostic Check Sheet* in Table 3 are computed as the ratio of the number of patients with a positive score for the given feature and the total patient count (first row) of the given subtype. As the *Diagnostic Check Sheet* features are absent in the MDS dataset, we do not include the summary statistics of the patients mapped in the MDS dataset. We define a distinguishable feature for a given subtype as a feature with a difference greater than 0.1 from the mean value of all patients (i.e., the corresponding value under the “Overall” column). We define a distinguishable feature for our proposed set of final subtypes as the feature that acts as a distinguishing feature for 50% or more subtypes in the set (in our case, it means at least three subtypes out of the six final subtypes).

### 4.6 Validation study with an independent PD cohort

We replicated the subtype identification in an independent PD cohort to test the generalizability and validity of our subtyping results. Here, we use the data of the first validation study of the Movement Disorder Society Non-Motor Rating Scale by Chaudhuri et al. (2020). This was an international multicenter cross-sectional study. English-speaking patients with a diagnosis of PD, as defined by the MDS criteria, were included. Exclusion criteria included Parkinsonism due to other neurodegenerative diseases or secondary causes, moderate or greater cognitive impairment (defined as a Montreal Cognitive Assessment (MoCA) score of < 21), and active medical or psychiatric disorders or treatment that precluded accurate assessments. Patients were recruited from six movement disorders units in England (*n* = 5) and the United States (*n* = 1) between October 2016 and September 2018. It was also previously used for the data-driven patient subtyping of PD patients by Rodriguez-Sanchez et al. (2021).

#### 4.6.1 Study population

MDS data comprises 402 patients with an average PD onset age of 59.23 ± 10.67 years, 62.19% are male, and the average PD duration is 8.2 ± 7 years. We remove subjects with juvenile-onset PD or an age of onset equal to fifty years. We use the same criteria as those for the LRRK2 data to identify patients with early and late onset PD. Thus, we obtained 321 and 68 patients with late onset and early onset PD, respectively. Here, we similarly observe a significant class imbalance as observed earlier for the LRRK2 data, skewed toward late onset PD patients. The resultant MDS dataset primarily comprises Stage 2 (54.5%) and 3 (27.25%) patients according to the Hoehn and Yahr Scale, followed by Stage 1 (13.11%) and Stage 4 patients (5.14%). The resultant MDS dataset has a few missing values. *Patient's clinical state (on/off)* has the highest missing percentage of 24.68%, followed by *Time since last dose of levodopa* with 22.11%, *Kinetic tremor—left hand* with 2.06%; two more features have between 1 to 2%, while the remaining features have less than 1% of missing data.

#### 4.6.2 Feature mapping between LRRK2 and MDS data

It is not straightforward and involves an intermediate feature mapping exercise. Table 5 shows the mapping at the level of individual features between LRRK2 and MDS datasets. However, the MDS dataset does not contain family history, genetic information (such as whether anyone biologically related to the patient has PD or carries a specific mutation, like the LRRK2 mutation), behavioral characteristics (including caffeine and smoking-related behaviors), and treatment response details.

## Data Availability

Publicly available datasets were analyzed in this study. The LRRK2 dataset used in this study can be accessed from the website of the Michael J. Fox Foundation for Parkinson's Research at: https://www.michaeljfox.org/news/lrrk2-cohort-consortium. The cleaned, imputed dataset used for training the machine learning classifier is available from the corresponding author on reasonable request. The dataset used for the validation study is available at https://github.com/ferjorosa/parkinson-subtypes/tree/main/data.
