# Supplementary material for: Decision tree-based approach to robust Parkinson's disease subtyping using clinical data of the Michael J. Fox Foundation LRRK2 cross-sectional study
Source: Front Artif Intell. 2025 Nov 21;8:1668206. doi: 10.3389/frai.2025.1668206 (PMC12678400; doi:10.3389/frai.2025.1668206)
Supplement: Supplementary file 1 [file Data_Sheet_1.pdf]

frontiers

Supplementary Material

1 SUPPLEMENTARY DATA

Sensitivity Analysis of PD classification regarding different age of onset-based cutoff values

We use the age of onset as 50 years to distinguish between EOPD and LOPD patients in this work, by following the recent study of *Mehanna et al.* Mehanna et al. (2022). However, we aim to understand the sensitivity of the PD classification model used to develop the candidate subtype rules to the *age of onset (AO)* criteria. Specifically, we analyze the effect of using different age of onset values of 45, 48, 52, and 55 years, instead of 50 years, for developing the decision tree-based PD classification model. On comparison of the top five features in terms of Gini importance score, we observe that three of them (*Geriatric Depression Scale Score*, *Time spent with dyskinesia*, and *Rest tremor amplitude - RUE* are present in four, three and two times respectively out of five cases) are robustly present in most of them, while the remaining two features of *Gait* and *Constipation Problems* are unique to the 'AO of 50' criteria. On the other hand, the test accuracy varies by a high margin between the five AO settings, with the highest accuracy of 0.705 being in the case of AO as 45, and the lowest accuracy of 0.553 being in the case of AO as 55. We further observe that the decision tree formed by the five AO criteria differs by a good margin, with the *Time spent with dyskinesia* feature being the most robust among them; we consider only the features present in the top two levels (starting from the root) of a decision tree. Therefore, we conclude that although the features are quite robust and consistent among the different AO settings, the learned decision tree is quite sensitive to the AO criteria used for constructing the target label of EOPD and LOPD patients for the PD classification model.

REFERENCES

Mehanna, R., Smilowska, K., Fleisher, J., Post, B., Hatano, T., Pimentel Piemonte, M. E., et al. (2022). Age cutoff for early-onset parkinson's disease: Recommendations from the international parkinson and movement disorder society task force on early onset parkinson's disease. *Movement Disorders Clinical Practice* 9, 869–878. doi:https://doi.org/10.1002/mdc3.13523
